# The association of *MTHFR* (rs1801133) with hypertension in an indigenous south African population

**DOI:** 10.3389/fgene.2022.937639

**Published:** 2022-07-22

**Authors:** Sihle E. Mabhida, Jyoti R. Sharma, Teke Apalata, Charity Masilela, Sibusiso Nomatshila, Lawrence Mabasa, Hannah Fokkens, Mongi Benjeddou, Babu Muhamed, Samukelisiwe Shabalala, Rabia Johnson

**Affiliations:** ^1^ Biomedical Research and Innovation Platform, SAMRC, Tygerberg, South Africa; ^2^ Department of Biotechnology, Faculty of Natural Science, University of the Western Cape, Cape Town, South Africa; ^3^ Division of Medical Microbiology, Department of Laboratory-Medicine and Pathology, Faculty of Health Sciences, Walter Sisulu University and National Health Laboratory Services, Mthatha, South Africa; ^4^ Department of Biochemistry, North-West University, Mmabatho, South Africa; ^5^ Division of Preventive Medicine and Health Behavior, Department of Public Health, Faculty of Health Sciences, Walter Sisulu University, Mthatha, South Africa; ^6^ Division of Infections Disease - University of Tennessee Health Sciences Center (UTHSC), Memphis, TN, United States; ^7^ Centre for Cardio-Metabolic Research in Africa, Division of Medical Physiology, Faculty of Medicine and Health Sciences, Stellenbosch University, Tygerberg, South Africa

**Keywords:** hypertension, methylenetetrahydrofolate reductase gene, MTHFR, single-nucleotide polymorphism, Africa, genetic variation

## Abstract

**Aims:** The current study sought to investigate the association between the methylenetetrahydrofolate reductase (*MTHFR*) variant (rs1801133) and the risk of developing hypertension (HTN) in an indigenous South African population.

**Methods:** A total of 442 participants (hypertensive, *n* = 279 and non-hypertensive, *n* = 163) from the indigenous tribe residing in Mthatha, Eastern Cape (South Africa) were recruited. HTN was defined as a systolic (SBP) and diastolic blood pressure (DBP) of ≥130/80 mmHg following American Heart Association guidelines. The genotyping of *MTHFR* (rs1801133) was assessed using MassARRAY^®^ System. Thereafter, the association between rs1801133 in various genetic models and HTN was determined by logistic regression model analysis. Furthermore, the interaction between rs1801133 and selected risk factors on HTN was performed using the open-source multifactor dimensionality reduction (MDR).

**Results:** The low frequency of the T allele (5%) was also observed when compared with the C allele (95%) in both cases and controls. After adjusting for confounding factors (gender, smoking status, BMI, and blood glucose levels), there were no significant associations were observed between rs1801133 and the risk of HTN in all genetic models: genotypic (OR 0.75, 95% CI 0.29–1.95, *p* = 0.56), dominant (OR 0.86, 95% CI 0.35–2.16, *p* = 0.75), co-dominant (OR 1.33, 95% CI 0.51–3.48, *p* = 0.55) and allelic (OR 0.80, 95% CI 0.49–1.62, *p* = 0.70) in logistic regression analysis. However, a significant interaction was reported among rs1801133, age, and gender (*p* < 0.0001) with the risk of HTN.

**Conclusion:** The present study reports on the lack of association between MTHFR (rs1801133) and the risk of HTN in an indigenous South African tribe. However, an interaction between gender, age, and rs1801133 was observed. Thus, future studies with a large sample size are required to further validate these findings.

## Introduction

Hypertension (HTN) is a multifactorial disorder with genetic, environmental, and demographic factors contributing to its prevalence and progression. Over the past years, numerous researchers have uncovered the significant role of inter-individual genetic variations in the development, progression, and control of the disease (Tuder et al., 2013; Fontana et al., 2014; Bayramoglu et al., 2015). Indeed, genome-wide association studies have successfully identified several genetic variants linked to the development and the progression of HTN (Padmanabhan et al., 2008; Li et al., 2012), including methylenetetrahydrofolate reductase (*MTHFR*) gene variants, with rs1801133 being the most common and widely studied variant located in this gene (Xi et al., 2013; Fan et al., 2016). However, their role in the pathophysiology of HTN particularly among individuals of African descent remains unclear (Amrani-Midoun et al., 2016; Ghogomu et al., 2016).

The *MTHFR* gene encodes methylenetetrahydrofolate reductase, a key regulatory enzyme in folate and homocysteine (Hcy) metabolism, and in the production of nitric oxide, a potent vasodilator that is important in blood pressure regulation (Goyette et al., 1994; McMahon et al., 2016). The rs1801133 polymorphism (677 C > T) is found on exon 4 of the *MTHFR* gene. The consequence of this polymorphism is a change of amino acid 222 from alanine to valine, leading to a decrease in *MTHFR* activity and an increase in plasma Hcy levels (Tawakol et al., 1997; Heux et al., 2004). Literature suggests that elevated plasma concentration of Hcy may injure the vascular endothelium, resulting in HTN (Tawakol et al., 1997; Heux et al., 2004). While other studies have shown that rs1801133 was associated with HTN in populations (Bouzidi et al., 2020; Wu et al., 2022).

Furthermore, these studies established that carriers of rs1801133 present with high levels of plasma Hcy and are more likely to develop HTN (Guenther et al., 1999; Wu et al., 2022). Whereas a reduction in *MTHFR* activity was linked with the loss of riboflavin, a cofactor associated with a decrease of Hcy among carriers of the *MTHFR* variant (Guenther et al., 1999; Yamada et al., 2001; McNulty et al., 2006). Most importantly, accumulative studies have suggested that rs1801133 polymorphism could be an independent risk factor for HTN in different ethnic groups (Heux et al., 2004; Ilhan et al., 2008; Nassereddine et al., 2015), and it has been associated with severe diastolic HTN in pregnant women (Kosmas et al., 2004). Even so, conflicting results have been obtained, particularly among African population studies (Al-Kordy et al., 2012; Nassereddine et al., 2015; Amrani-Midoun et al., 2016; Ghogomu et al., 2016). Thus, several authors have emphasized the need to conduct more African studies to further validate the role of *MTHFR* polymorphisms in the pathogenesis of HTN (Amrani-Midoun et al., 2016; Ghogomu et al., 2016; Olczak et al., 2021).

Undoubtedly, HTN as a chronic medical condition, has contributed significantly to the rapid rise in global premature deaths, especially among Black African adults (Adeniyi et al., 2016; Sharma et al., 2021). Moreover, the devastating outcome has been the limited data currently informing on the incidence and pathological mechanisms driving the onset and progression of this medical condition among black African adults (Mabhida et al., 2021). Studying the genetics of HTN can revolutionize our understanding the pathophysiology of the disease and the discovery of effective treatment strategies. Importantly, our research group has actively explored this research niche within an indigenous South African population. Our group has demonstrated a high prevalence (75%) of HTN in the village of Mthatha, Eastern Cape (Adeniyi et al., 2016; Sharma et al., 2021). We further conducted a narrative synthesis of the literature that predicted a possible association between *MTHFR* rs1801133 and the development of HTN among individuals of African origin (Mabhida et al., 2022). Our findings highlighted the necessity of performing genomic studies on indigenous African individuals, as very few studies have addressed this research niche in a purely black African population. Thus, this study aimed to investigate the association between *MTHFR* (rs1801133) and HTN in the indigenous South African population of Mthatha in the Eastern Cape province of South Africa. Additionally, the study aimed to explore the interaction between single nucleotide polymorphism (SNP) and sociodemographic as well as their interaction with the risk of HTN.

## Material and methods

### Ethical clearance

The ethical approval for this study was provided by the Senate Research Committee of the University of the Western Cape (Ethics clearance number BM19/8/19), Water Sisulu University (073/15), and the South African Medical Research Council (EC028-8/2020). The study conforms to the ethical guidelines of the Declaration of Helsinki (Puri et al., 2009). Written informed consent in both English and IsiXhosa was obtained from all the study participants.

### Power and sample size estimation

The appropriate sample and power size were estimated using the Power and Sample Size Program (PS version 3.1.2). The study initially planned to recruit approximately 430 participants (1:1 ratio). “Previous data indicate that the probability of exposure among controls is 0.75. If the true odds ratio for disease in exposed subjects relative to unexposed subjects is 2, we will need to study 215 case patients and 215 control patients to be able to reject the null hypothesis that this odds ratio equals 1 with a probability (power) 0.8. The Type I error probability associated with this test of this null hypothesis is 0.05 (Dupont and Plummer, 1990; Dupont and Plummer, 1998)”.

### Study design and patient selection

A total of 442 participants belonging to the indigenous Xhosa tribe in the Eastern Cape province of South Africa, aged 18 years and above were recruited from four districts (OR Tambo, Alfred Nzo, Chris Hani, and Joe Gqabi). Participants were divided into two groups 1) the non-hypertensive group (*n* = 163), and 2) hypertensive group (*n* = 279). HTN was defined as average SBP or DBP of ≥130/80 mmHg or using at least one class of antihypertensive medication following the American Heart Association guidelines (AHA) (Whelton et al., 20172018). Blood pressure values were measured 3 consecutive times, after 10 min of seated rest before the first measurement and 5 min intervals between each measurement with a manual aneroid sphygmomanometer. SBP and DBP were determined by the first and the fifth Korotkoff sounds. The average of three consecutive measurements to the nearest 2 mmHg was recorded, with a time interval of at least 2 min. Participants were selected in general consultation in the same centres.

### Data collection and sampling procedure

The data records were collected using the World Health Organization (WHO) STEPwise questionnaire which was uploaded onto the Research Electronic Data Capture (REDCap), a web-based application for building and managing online surveys and databases. Data collected included information on environmental or sociodemographic factors (smoking, age, gender), anthropometric measurements (body mass index, BMI), and blood glucose levels. All selected variables were defined as previously described by Sharma et al. (2021) (Sharma et al., 2021) Venous blood (5 ml) was collected in EDTA tubes. The samples were stored at −80°C until used. A blood sample was also taken after an overnight fast to measure the glucose levels.

### Genomic DNA isolation and genotyping

Peripheral blood samples were collected from all subjects and the DNA was extracted using a QIAamp DNA Blood Midi kit (Qiagen, Valencia, CA, United States) as per the manufacturers’ instructions. DNA quantity and quality were determined using a Nano-Drop™ 2000/2000c UV/VIS Spectrophotometer (ThermoScientific™). Thereafter, the presence of *MTHFR* (rs1801133) was analyzed using MassARRAY^®^ System (Agena Bioscience™).

### Statistical analysis

Statistical analyses were performed using Stata/IC version 17.0 (Stata Corp, United States). The general characteristics of the participants were expressed as frequency (percentages). A chi-square test (non-parametric) was used to evaluate the genotypic distribution of the *MTHFR* (rs1801133) variant. Logistic regression analysis was used to examine the potential effects of the selected SNP (rs1801133) on the risk of developing HTN under various genetic models (genotypic, dominant, recessive, co-dominant, and allelic models) (Fan et al., 2016). A method suggested by Thakkinstian et al. (2005) was used to select the most appropriate genetic model. Multivariate regression analysis was carried out between rs1801133 variant and independent variables such as age, gender, smoking status, BMI and blood glucose levels. The probability of exposure given the outcomes (OR) and their 95% confidence intervals (CI) was used to present the association between the SNP and the risk of HTN. A two-tailed statistical significance was evaluated by using a *p*-value of <0.05. Interactions between rs1801133 and selected environmental factors on HTN were detected using the open-source multifactor dimensionality reduction (MDR) software package version 3.0.2. Minor allele frequency (MAF) and Hardy–Weinberg equilibrium (HWE) tests were calculated using Genetic Analysis in Excel (GenAIEx) Version 6.5, where a *p*-value < 0.05 indicated a deviation from the Hardy Weinberg principle.

## Results

### General characteristics of the study cohort

The present study sampled 442 individuals over the age of 18 years, of whom 19% (*n* = 82) were males and 81% (*n* = 360) were females. The mean for age was 46.96 ± 14.55 years. The distribution of baseline characteristics of categorical variables (age, gender, smoking status, BMI, and blood glucose levels) is summarized in Table 1.

**TABLE 1:** General characteristics of the study cohort.

Variable	Total *n* = 442, *n* (%)	Hypertensive *n* = 279, *n* (%)	Non-hypertensive *n* = 163, *n* (%)	*p-*values
Age (years)				<0.001
18–35 years	109 (25)	51 (18)	58 (36)	
36–49 years	146 (33)	93 (33)	53 (33)	
50–64 years	132 (30)	93 (33)	39 (24)	
≥65 years	55 (12)	42 (16)	13 (7)	
Mean age (years)				
18–35 years	28.83 ± 4.45	28.90 ± 4.45	28.77 ± 4.51	
36–49 years	42.55 ± 3.96	42.64 ± 3.83	42.39 ± 4.20	
50–64 years	56.21 ± 3.65	56.07 ± 3.67	56.56 ± 3.62	
≥65 years	72.38 ± 5.60	72.61 ± 5.84	71.61 ± 4.89	
Gender				0.035
Female	360 (81)	235 (84)	125 (77)	
Male	82 (19)	44 (16)	38 (23)	
Smoking Status				0.010
Yes	31 (7)	13 (5)	18 (11)	
No	404 (93)	263 (95)	141 (89)	
Body Mass Index				<0.001
Normal	116 (27)	57 (20)	59 (41)	
Overweight	105 (25)	65 (23)	40 (25)	
Obese	207 (48)	156 (57)	51 (34)	
SBP (mm Hg)	130.79 ± 20.59	140.02 ± 18.93	113.72 ± 9.82	<0.001
DBP (mm Hg)	83.46 ± 11.56	89.24 ± 9.74	72.78 ± 5.42	<0.001
Blood glucose levels				0.034
Normal - FBS <5.6 mmol/L	259 (59)	163 (59)	96 (59)	
Prediabetic - FBS >5.6–6.9	85 (19)	59 (21)	26 (16)	
Diabetic - FBS >7 mmol/L	98 (22)	57 (20)	41 (25)	

Chi-square tests of association were conducted; significance is determined at *p* < 0.05 (overall, for a given parameter).

### Correlation between covariates and hypertension susceptibility

In Table 2, summarize the ORs with corresponding 95% CIs for the association of environmental factors (age, gender, smoking status, BMI, and blood glucose levels) with HTN using univariate analyses. In the unadjusted model, the logistic regression analysis showed a significant association with older age (36 to ≥65) (*p* = 0.007 and *p* < 0.001 respectively), being male (*p* = 0.034), smoking habits (*p* = 0.012), obese (*p* < 0.001), and prediabetic/diabetic (*p* = 0.031) with the increasing risk of developing HTN.

**TABLE 2:** Associations of age, gender, BMI, smoking status, and blood glucose levels with the risk of developing HTN.

Variable	Hypertensive	Non-hypertensive	Crude odds ratios (95%CI)	*p*-Value
Age (years)				
18–35 years	51	58	1.0*	
36–49 years	93	53	1.99 (1.20–3.31)	0.007
50–64 years	93	39	2.71 (1.59–4.60)	<0.001
≥65 years	42	13	3.67 (1.77–7.60)	<0.001
Gender				
Female	235	125	1.0*	
Male	44	38	1.69 (1.03–2.78)	0.034
Smoking Status				
No	13	18	1.0*	
Yes	263	141	0.39 (0.18–0.81)	0.012
Body Mass Index				
Normal	57	59	1.0*	
Overweight	65	40	1.68 (0.98–2.87)	0.057
Obese	156	51	3.16 (1.95–5.12)	<0.001
Blood glucose levels				
Normal - FBS <5.6 mmol/L	163	96	1.0*	
Prediabetic - FBS >5.6–6.9	59	26	0.73 (1.01–2.96)	0.065
Diabetic - FBS >7 mmol/L	57	41	1.39 (2.11–5.45)	0.031

*p-Value < 0.05 was considered statistically significant.

BMI, body mass index; CI, confidence interval.

### Association between *MTHFR* (rs1801133) polymorphism and hypertension susceptibility

Table 3 shows the genotypic and allelic frequencies for the *MTHFR* (rs1801133) variant and the risk of developing HTN under genotypic, dominant, recessive, co-dominant, and allelic. The most frequently observed genotype was the homozygous CC (hypertensive, 91% and non-hypertensive, 89%) followed by heterozygous CT (hypertensive, 8% and non-hypertensive, 11%). The homozygous genotype TT had the lowest frequency in the studied population (hypertensive, 1% and non-hypertensive, 0%). Furthermore, the low frequency of the T allele of rs1801133 (5%) was also observed in both hypertensive and non-hypertensive individuals when compared with the C allele (95%). After testing for Hardy-Weinberg principle using *X* ^2^ and *p* < 0.05, all alleles and genotypes were in agreement with the hypothesis (*p* = 0.069).

**TABLE 3:** Association of the *MTHFR* (rs1801133) polymorphism with risk of developing HTN.

SNP	Hypertensive (*n*; %)	Non-hypertensive (*n*; %)	Crude odds ratios (95%CI)	*p*-Value	Adjusted odds ratios (95%CI)	*p*-Value
**rs1801133** (HWE, *p* = 0.069)						
			Genotypic			
CC	(254; 91)	(144; 89)	1.0*		1.0*	
CT	(21; 8)	(18; 11)	0.64 (0.33–1.25)	0.19	0.75 (0.29–1.95)^a^	0.56
TT	(3; 1)	(0; 0)	-	-	-	-
			Dominant			
CC	(254; 91)	(144; 89)	1.0*		1.0*	
CT + TT	(24; 9)	(18; 11)	0.75 (0.40–1.43)	0.34	0.86 (0.35–2.16)^a^	0.75
			Recessive			
CC + CT	(275; 99)	(162; 100)	1.0*		1.0*	
TT	(3; 1)	(0; 0)	-	-	-	-
			Co-dominant			
CC + TT	(18; 8)	(22; 11)	1.0*		1.0*	
CT	(144; 92)	(256; 89)	1.48 (0.72–2.84)	0.24	1.33 (0.51–3.48)^a^	0.55
			Allelic			
C	(529; 95)	(306; 95)	1.0*			
T	(27; 5)	(18; 5)	0.86 (0.47–1.60)	0.65		

a adjusted for age, gender, smoking status, BMI and blood glucose levels.

*p-Value > 0.05 was considered statistically not significant.

HWE, Hardy-Weinberg Equilibrium; MTHFR, methylenetetrahydrofolate reductase; BMI, body mass index; CI, confidence interval.

In the univariate analysis, the *MTHFR* rs1801133 variant was not significantly associated with HTN under various inheritance models [genotypic (OR = 0.75, 95% CI = 0.29–1.95, *p* = 0.56), dominant (OR = 0.86, 95% CI = 0.35–2.16, *p* = 0.75), recessive (*N/A*), co-dominant (OR = 1.33, 95% CI = 1.51–3.48, *p* = 0.55) and allelic models (OR = 0.80, 95% CI = 0.49–1.62, *p* = 0.70)]. After adjusting for confounding factors such as age, gender, smoking status, BMI, and blood glucose levels, the direction of association did not change (*p* > 0.05).

**FIGURE 1:**
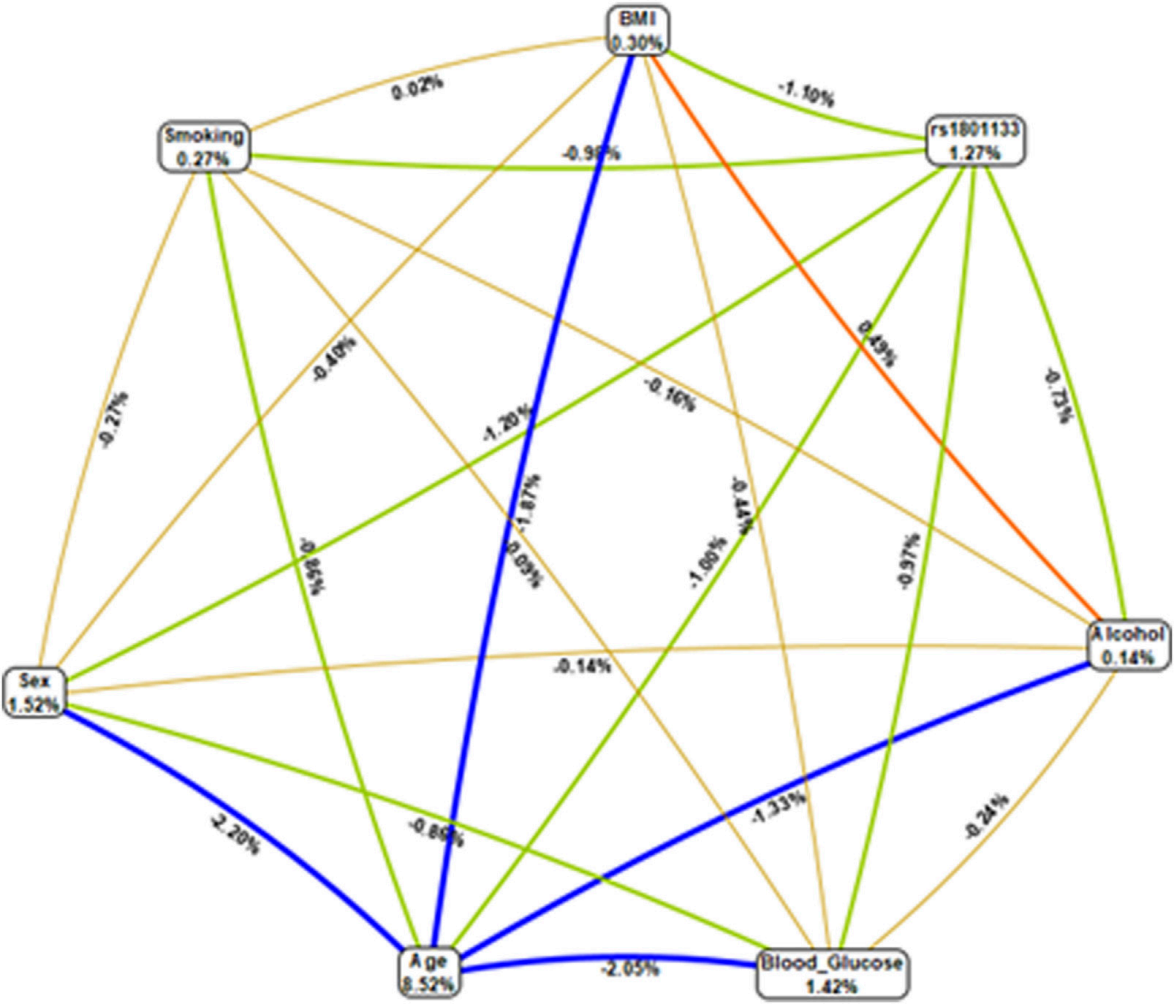
Multifactor dimensionality reduction combined attribute network demonstrating all possible interactions between rs1801133 and various environmental factors. Four different colours and various interactions between the genotypes of *MTHFR* (rs1801133) and selected variables (age, gender, smoking status, BMI, and blood glucose levels) were observed, and each colour represents a possible interaction. The width of the line indicates the strength of the interaction, whereas the thin lines represent weak interactions. Figures <1 and thin lines represent weak interactions. The strongest interactions are represented by figures ≥1 and thick lines. The image was generated using the open-source MDR software package version 3.0.2.

Epistatic interactions between the genotypes of *MTHFR* (rs1801133) and selected variables (age, gender, smoking status, BMI, and blood glucose levels) were assessed using multifactor dimensionality reduction (MDR) as presented in Table 4. The combination of age and rs1801133 had a high CVC score (9/10), however; the model was not significantly associated with HTN (*p* = 0.054). On the other hand, the combination of age, rs1801133, and gender (CVC = 7/10) showed a significant association with the risk of HTN (*p* < 0.0001). Other possible interactions are shown in Figure 1 **.**

**TABLE 4:** Interaction models between genetic and sociodemographic factors.

Variable	CV training score	Testing score	CVC	*p-*values
Age	0.6574	0.6574	10/10	<0.0001
Age, rs1801133	0.678	0.6648	9/10	0.054
Age, rs1801133, Gender	0.6973	0.6593	7/10	<0.0001

## Discussion

Previous data from our group demonstrated a high prevalence (75%) of HTN in the villages of Mthatha, Eastern Cape (Adeniyi et al., 2016; Sharma et al., 2021). Through a narrative synthesis of the literature, we further reported that there is a lack of African-specific data with regards to the association between *MTHFR* rs1801133 and HTN (Mabhida et al., 2021; Mabhida et al., 2022); thus, highlighting the significance of the current report. The present study aimed to investigate the association between rs1801133 and HTN in an indigenous South African population from the Eastern Cape province.

The current study reports on the lack of association between *MTHFR* (rs1801133) and the risk of HTN among an indigenous South African population (*n* = 442). Our findings are consistent with results reported by Amin et al. (Al-Kordy et al., 2012), and Amrani-Midoun *et al.* (Amrani-Midoun et al., 2016), where they also observed no association between rs1801133 and the risk of developing HTN in a cohort of Egyptian (*n* = 181) and Algerian (*n* = 154) adults, respectively. In contrast, a study done by Ghogomu *et al.* (Ghogomu et al., 2016) showed a significant association between rs1801133 and HTN among a native Bantu ethnic group of the South-West region of Cameroon (*n* = 91). Furthermore, Nasseredine et al. (Nassereddine et al., 2015) demonstrated that individuals of Moroccan (*n* = 203) origin who were homozygous carriers of rs1801133 were more likely to develop HTN.

Several studies performed in non-African countries also demonstrated that genotype TT or T allele of rs1801133 may increase the risk of HTN (Fan et al., 2016; Bouzidi et al., 2020; Wu et al., 2022). The discrepancies observed between these studies may be due to geographical location, ethnicity, and epigenetic mechanisms that are involved in the regulation of *MTHFR* gene expression (Fu et al., 2019; Meng et al., 2021). Suggesting that data linking HTN to rs1801133 remains controversial. Li et al. (Li et al., 2005) and Rosenberg *et al.* (Rosenberg et al., 2002) showed that the frequency of the T allele varies across different populations. The authors further demonstrated that individuals of African origin presented a low frequency of the T allele (<10%) in comparison to other ethnic groups (Shi et al., 2003; Guéant-Rodriguez et al., 2006; Romero-Sánchez et al., 2015). This was further confirmed by a study conducted by Atadzhanov et al. (Atadzhanov et al., 2014), who also observed a low frequency of the T allele in a Zambian population. In agreement with the later studies (Atadzhanov et al., 2014), our study demonstrated that the T allele and the TT genotype frequencies of *MTHFR* (rs1801133) were lower (5%) than the C allele (95%) in both hypertensive and non-hypertensive individuals. We, therefore, tested for Hardy-Weinberg equilibrium, and none of the genetic models deviated from the principle. This is an indication that the genotype and allele frequencies in our study population remained constant between generations, and that there was no random mating that occurred. Our findings further suggest that a positive association between *MTHFR* (rs1801133) polymorphism and HTN among African individuals may not be strong enough to withstand statistical interrogation by false-positive report probability (Meng et al., 2021). Of note, the current study as well as the reference studies (Al-Kordy et al., 2012; Nassereddine et al., 2015; Amrani-Midoun et al., 2016; Ghogomu et al., 2016), were composed of relatively small sample sizes; thus, the observations made remain to be explored in future studies with larger sample sizes.

Some observational studies have shown that genetic changes are inseparable from the impact of environmental factors such as diet, exercise, smoking, and drinking (Anand, 2005; Zhong et al., 2017). As a result, SNP-environment interaction could not be ignored as our existing understanding of the pathogenesis of HTN indicates an interplay between genetic and environmental factors (Fan et al., 2016). Although no association was established between rs1801133 and HTN in the present study, an interaction between rs1801133, age, and gender was observed, suggesting a possible synergistic effect. This was in agreement with the previous studies which also reported that rs1801133, aging, and gender are potential risk factors for elevated Hcy levels (Ma et al., 2020), decreased uric acid (Massie et al., 1991), and folate deficiency (James et al., 2002), which increases the risk of HTN and hyperhomocysteinemia (Nygård et al., 1995; Refsum et al., 2006). Furthermore, Boers et al. (Boers et al., 1985), and Ueland et al. (Ueland et al., 1993), have demonstrated that premenopausal women have lower Hcy levels than men and postmenopausal women (Nygård et al., 1995; Refsum et al., 2006). The reasons for the higher Hcy concentrations at older ages are not well understood, although changes in renal function are certainly involved. Higher Hcy concentrations in men in comparison to women may be explained by differences in muscle mass, hormone, and vitamin status (Refsum et al., 2004). Thus, this suggests that the interaction between age, gender, and the *MTHFR* (rs1801133) polymorphism in HTN may be partially due to their interactive effects on Hcy concentrations and DNA methylation status (Selhub et al., 1993; Kotchen, 2010; Kato et al., 2015; Liu et al., 2015; Fan et al., 2016). But this hypothesis needs to be further explored in a bigger cohort.

### Limitations and strengths of this study

It is noteworthy to mention that several limitations need to be considered in interpreting the findings of our study. Firstly, the sample size of our study was relatively small, of which could very likely introduce random errors and bias, resulting in unreliable results. However, this limitation projects another strength as it is often better to test a new research hypothesis on a small number of participants before progressing to a large number. This is especially important not to conserve resources. Importantly, our findings will be a stepping-stone to larger ongoing clinical and genetic studies of HTN in the South African population. Notably, also highlights another strength of the current report, as very few studies have addressed this research niche in a purely black African population. Importantly, unlike other reports (Nassereddine et al., 2015; Ghogomu et al., 2016), our study did perform an adjustment for confounding factors such as gender, age, and smoking status. This indicates an urgent need to carefully plan African-specific studies with large sample sizes to be able to draw conclusions on the association between rs1801133 and HTN. Secondly, this study used a cross-sectional design, which precludes causal inference, consistent with recruiting participants from only one ethnic group (Xhosa population of Mthatha, Eastern Cape). Further highlights that our findings are generalised to the included study population and individuals residing in similar settings in the province. Lastly, the current project was planned to recruit approximately 430 participants, with a 1:1 ratio of cases to controls. But, due to the high prevalence of the HTN (75%) in our study population, a high number of hypertensive patients were included in the study (*n* = 279). However, the T allele frequency of study population was not influenced by this limitation as it remained low (5%). Despite these limitations, to the best of our knowledge, this is the first study to investigating the association between the *MTHFR* (rs1801133) variant and HTN in South Africa, and further explored a unique and important feature of SNP-environment interaction. Overall, these findings remain important in addressing the limited data on the *MTHFR* variant (rs1801133) and the risk of developing HTN in indigenous populations of South Africa.

## Conclusion

In the present study, we demonstrated the lack of association between *MTHFR* (rs1801133) and HTN among individuals of Xhosa origin. However, a potential synergistic effect of the rs1801133 with age and gender on HTN susceptibility was observed. The current study has laid a foundation for future studies by highlighting the significance of considering the impact of environmental factors in genetic association studies. Furthermore, future studies with a large sample size are required to further validate our findings.

## Data Availability

The original contributions presented in the study are included in the article/supplementary materials, further inquiries can be directed to the corresponding author.
